# Worldwide Evolution of Vaccinable and Nonvaccinable Viral Skin Infections: Google Trends Analysis

**DOI:** 10.2196/35034

**Published:** 2022-10-04

**Authors:** Thierry Simonart, Xuân-Lan Lam Hoai, Viviane de Maertelaer

**Affiliations:** 1 Department of Dermatology Delta Hospital, Centre Hospitalier Interrégional Edith Cavell Université Libre de Bruxelles Brussels Belgium; 2 Department of Dermatology St Pierre - Brugmann - Hôpital Universitaire des Enfants Reine Fabiola University Hospitals Université Libre de Bruxelles Brussels Belgium; 3 Department of Biostatistics Institut de Recherche Interdisciplinaire en Biologie Humaine et Moléculaire Université Libre de Bruxelles Brussels Belgium

**Keywords:** big data, infodemiology, measles, varicella, rubella, hand, foot, mouth disease, skin infection, epidemic, wart, skin, dermatology, trend, Google search, web search, surveillance, vaccinable, incidence, viral epidemics, distribution, measles

## Abstract

**Background:**

Most common viral skin infections are not reportable conditions. Studying the population dynamics of these viral epidemics using traditional field methods is costly and time-consuming, especially over wide geographical areas.

**Objective:**

This study aimed to explore the evolution, seasonality, and distribution of vaccinable and nonvaccinable viral skin infections through an analysis of Google Trends.

**Methods:**

Worldwide search trends from January 2004 through May 2021 for viral skin infections were extracted from Google Trends, quantified, and analyzed.

**Results:**

Time series decomposition showed that the total search term volume for warts; zoster; roseola; measles; hand, foot, and mouth disease (HFMD); varicella; and rubella increased worldwide over the study period, whereas the interest for *Pityriasis rosea* and herpes simplex decreased. Internet searches for HFMD, varicella, and measles exhibited the highest seasonal patterns. The interest for measles and rubella was more pronounced in African countries, whereas the interest for HFMD and roseola was more pronounced in East Asia.

**Conclusions:**

Harnessing data generated by web searches may increase the efficacy of traditional surveillance systems and strengthens the suspicion that the incidence of some vaccinable viral skin infections such as varicella, measles, and rubella may be globally increasing, whereas the incidence of common nonvaccinable skin infections remains stable.

## Introduction

Viral skin infections are common reasons for consulting health care providers and represent a substantial public health concern throughout the world. Effective vaccines against viral diseases with primary or prominent cutaneous manifestations include those directed against measles and rubella (now commonly used together with a mumps vaccine as the trivalent MMR), human papillomavirus, varicella, and zoster [1]. Although vaccination is an effective method of preventing these diseases [2,3], large-scale epidemiologic data on their evolution and distribution remain scarce. Studying the population dynamics of these viral epidemics using traditional epidemiological methods is costly and time-consuming, especially over wide geographical areas. Moreover, traditional studies may be affected by the underreporting of cases, data quality issues, reporting delays, or even conflicts of interest, resulting in missed opportunities to respond to trends in disease prevalence. Over the last 2 decades, the use of the internet as an initial information source has become almost ubiquitous among the general population. Google Trends is a web-based tracking system by Google that analyzes the popularity of top search queries in the Google search engine across various regions and languages [4,5]. Since 2004, Google Trends has been increasingly used to explore web-based health-seeking behaviors, offering a new, interesting tool to monitor public attention with regard to specific diseases. The association between the predictive power of Google Trends and the data of official surveillance systems has been studied for a wide range of medical topics, with the conclusion that it can provide useful real-time data about epidemiological surveillance, screening, and treatment options [6-13]. Previous studies have examined the utility of Google Trends to monitor various infectious diseases such as influenza, tuberculosis, Lyme disease, COVID-19, dengue, genital warts, lice, or scabies [6-13]. Since the possible resurgence of some viral skin diseases is a growing concern [14], we investigated whether Google Trends could reflect possible changes in the epidemiology of cutaneous viral infections.

## Methods

### Google Trends Data

The data were obtained from Google Trends, a public web-based database and analytics tool of Google Inc [4,5]. Similar applications of infoveillance in the investigation of health campaign effectiveness have been described previously [15-17].

Google Trends generates data and allows the user to compare the relative search volume (RSV) of 2 or more search terms, offering temporal and geographic models based on the specific terms [16-18]. It shows how often a given search term is entered into the Google search engine relative to the website’s total search volume over a given period of time. Google Trends can be used for comparative keyword research and to discover event-triggered spikes in keyword search volume.

The RSV is assigned to the search terms. The RSV values represent the goal of the research based on the highest point of the plot with respect to a region or a specific period. The RSV values do not represent absolute search volume numbers but rather normalized values reflected on a scale from 0 to 100, where 100 is the point of maximum popularity among the search terms or topics over a specified time frame. When no sufficient data are found regarding the search term, the score drops to 0 [5,12,13,19,20]. Relative monthly scores for all search terms and topics are expressed as relative interest scores, which are surrogates for the relative popularity of a particular search term and topic over that time frame.

The keywords were selected from articles on viral skin diseases or infections [21,22], and the usual name of the most common diseases were considered.

A search-term query in Google Trends provides searches for an exact search term, whereas a topic query includes related search terms (in any language, such as German, Portuguese, Chinese, Ukrainian, or Spanish) [23]. Google Trends facilitates the easy comparison of the given terms regardless of the language of Google users. We focused our analysis on the “Related Searches” section, which shows queries (and not keywords) that are related to the entered terms (which are instead true keywords). The data were obtained using the following topic queries, in the “Global” category (all available categories in Google Trends were included): “rougeole” (“measles” in French) as the subject; “herpès” (“herpes” in French) as the disease; “varicelle” (“chickenpox” in French) as the disease; “zona” (“zoster” in French) as the subject; “syndrome pieds-mains-bouche” (“hand, foot, and mouth disease” [HFMD] in French) as the disease; “molluscum contagiosum” as the subject; “verrue” (“warts” in French) as the subject; “pytiriasis rosé de Gibert” (“*Pityriasis rosea*” in French) as the disease; “exanthème subit” (“roseola” in French) as the disease; and “rubéole” (“rubella” in French) as the disease. The data were obtained in the time frame elapsing from January 1, 2004, to May 2021 (n=209 months) worldwide and aggregated by month. To compare the temporal evolution of the searches, the file in CSV format for each search was downloaded separately.

### Ethical Considerations

Ethics approval for this type of study was not required as none of the queries in the Google database can be associated with any identity or physical location, as specified in Google’s privacy policy [24].

### Data and Statistical Analysis

For the entire period (n=209 months), linear adjusted lines of the RSV index representing a normalized value, ranging from 0 (no searches) to 100 (for the peak of the search), were generated separately for several variables of interest. These linear fitted lines visually compared the trends of interest in common viral skin infections over the past 17 years. Seasonality was investigated through decomposition time series multiplicative models [25]. They were used with 12 months as the number of seasons on the RSV index (dependent variable). The quality of the model was evaluated through the pseudo *R*-squared for each variable. Seasonality was investigated for each variable. Their amplitudes, quantified as the difference between their highest and lowest monthly coefficients, were compared. Technical details concerning the statistical model and analysis are described in Multimedia Appendix 1. The statistical software used were SPSS (version 27.0; IBM Corp) and NCSS (version 10; NCSS LLC).

## Results

**Figure 1:**
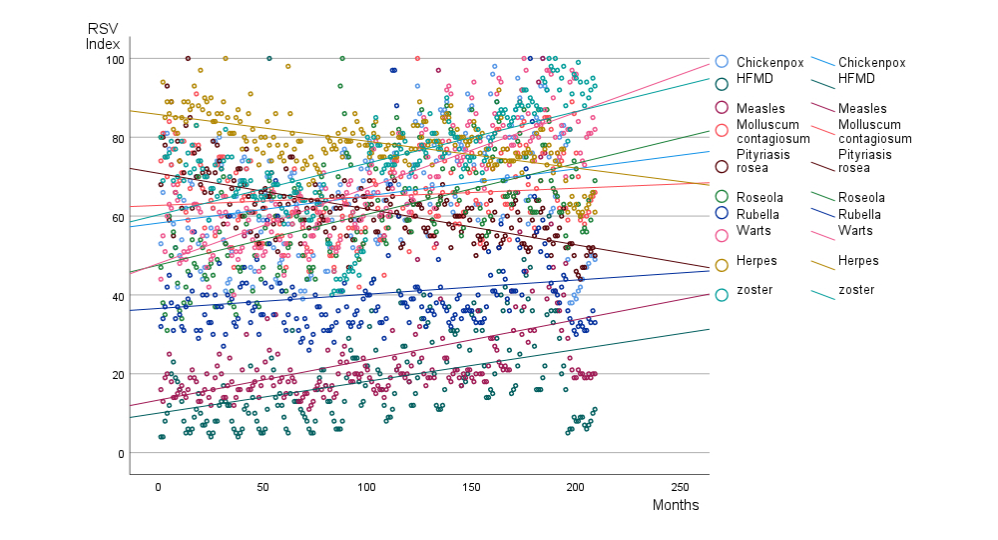
Data corresponding to searches for chickenpox; herpes; hand, foot, and mouth disease (HFMD); measles; molluscum contagiosum; *Pityriasis rosea*; roseola; rubella; warts; and zoster from Google Trends time data (17 years; 209 months). To compare the temporal evolution of the searches, data for each search were downloaded separately and are presented as a relative search volume (RSV) index. They do not represent absolute search volume numbers but rather a normalized value, ranging from 0 (for no searches) to 100 (for the peak of the search). The linear trends are represented.

The temporal evolution for the worldwide 17-year Google Trends data (from January 2004 to May 2021) regarding the variables mentioned under the Methods section is presented in Figure 1. They were adjusted through linear straight lines showing that the total search term volume for chickenpox, HFMD, measles, molluscum contagiosum**,** roseola, warts, and zoster increased worldwide over the study period, whereas the interest for *Pityriasis rosea* decreased. We found nearly no change (slopes ≤0.022) in interest for molluscum contagiosum (Table 1). The quality of the adjustment to the multiplicative model was satisfying.

**Figure 2:**
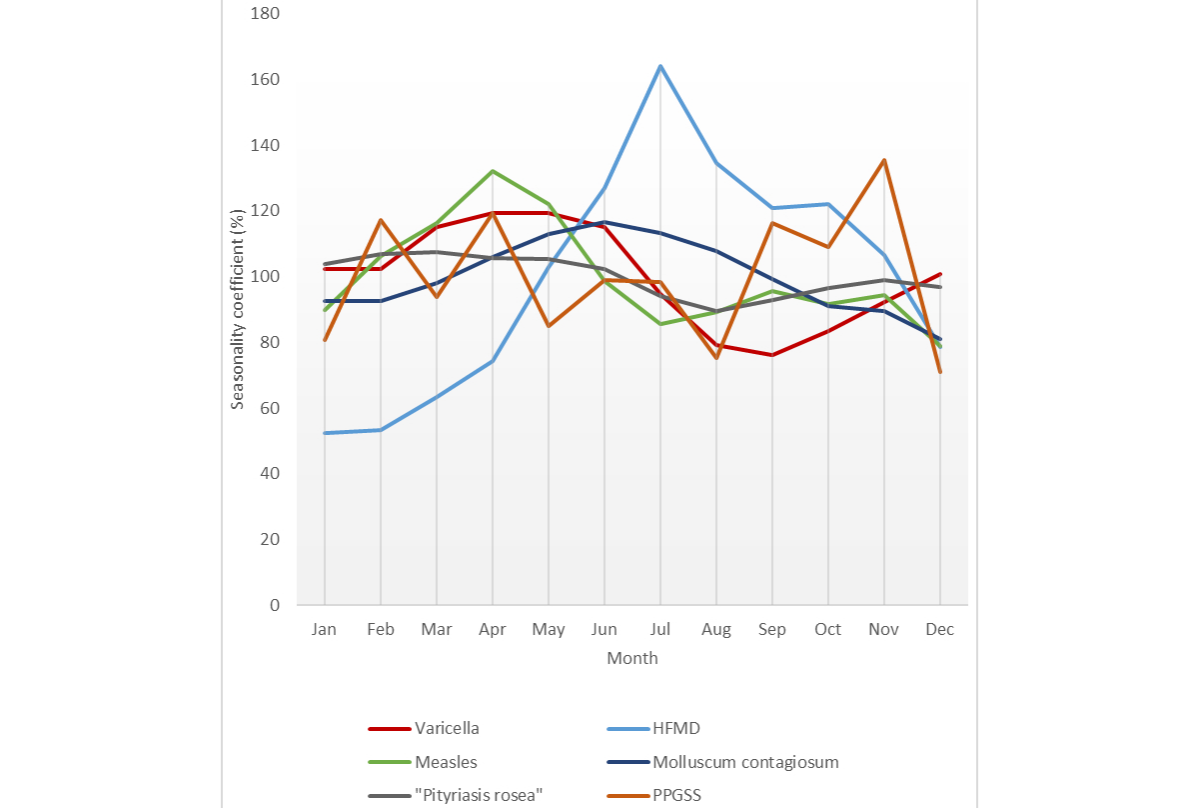
Seasonality coefficients (moving averages) of varicella, HFMD, measles, molluscum contagiosum, and *Pityriasis rosea*. For the clarity of the graph, only the infections with the highest seasonality coefficients are represented. HFMD: hand, foot, and mouth disease; PPGSS: papular-purpuric gloves and socks syndrome.

Seasonality in worldwide internet searches (reflecting mainly the Northern Hemisphere, since 90% of the world’s population and most of the internet users lives there [26,27]) was quantified as the difference between the highest and lowest seasonality coefficients, which were in decreasing order as follows: HFMD, chickenpox, measles, molluscum contagiosum, warts, roseola, rubella, *Pityriasis rosea*, zoster, and herpes (Table 1). The peaks of interest were in March for *Pityriasis rosea*; April for measles and chickenpox; May for rubella; June for molluscum contagiosum; July for HFMD, roseola, zoster, and warts; and August for herpes and zoster (Table 1 and Figure 2).

The top 5 regions where the queries for measles and rubella were the most popular were exclusively African countries. The top 5 regions where the queries for HFMD and roseola were the most popular were mostly in East Asia (Table 2).

**Table 1:** Seasonality and trends in worldwide internet searches for varicella, herpes, HFMD, measles, molluscum contagiosum, *Pityriasis rosea*, Roseola, rubella, warts, and zoster.

		Varicella	Herpes	HFMD^a^	Measles	MC^b^	*Pityriasis rosea*	Roseola	Rubella	Warts	Zoster
**Month, seasonal coefficient (%)**											
	January	103	100	55	89,8	93	103	85	91	92	97
	February	105	103	58	113	93	108	91	100	93	96
	March	116	100	67	114	98	107	92	108	95	98
	April	118	100	82	123	106	105	105	113	101	100
	May	117	99	120	125	112	104	116	115	107	100
	June	113	97	122	98	115	104	120	101	111	104
	July	95	103	156	84	113	94	124	92	116	105
	August	79	105	132	90	108	90	110	96	114	105
	September	77	99	115	95	99	93	85	99	102	102
	October	84	98	116	90	91	97	91	102	94	101
	November	93	99	101	93	90	98	85	98	90	98
	December	101	97	75	77	82	97	84	86	84	93
Delta		41	8	101	51	33	18	40	29	32	12
Slope of the adjusted straight lines		–0.091	–0.112	–0.024	0.015	–0.146	–0.121	0.104	–0.016	0.137	0.099

^a^HFMD: hand, foot, and mouth disease.

^b^MC: molluscum contagiosum.

**Table 2:** Top 5 regions for the queries for HFMD, measles, *Pityriasis rosea*, roseola, and rubella.

Viral skin infection	Region				
	Rank 1	Rank 2	Rank 3	Rank 4	Rank 5
HFMD^a^	China	Singapore	Vietnam	Finland	Hong Kong
Measles	Niger	Chad	Congo-Kinshasa	Madagascar	Guinea
*Pityriasis rosea*	Jamaica	Bulgaria	Belarus	Ukraine	Russia
Roseola	Vietnam	Taiwan	Japan	Czechia	Hong Kong
Rubella	Cameroon	Benin	Algeria	Ivory Coast	Congo-Brazzaville

^a^HFMD: hand, foot, and mouth disease.

## Discussion

Although some childhood viral diseases such as measles, rubella, or varicella are notifiable diseases [28], others such as warts, molluscum contagiosum, roseola, HFMD, or *Pityriasis rosea* are not reportable conditions in most countries, so studies on their changing trends and geographic distribution remain scarce.

Google Trends, a web-based tracking system of internet search volumes, has been extensively used in the field of infectious diseases, both for monitoring and surveillance purposes [6-13,29], and has been shown to be truly reliable for the prediction of epidemic outbreaks [9-12].

Previous data indicate a significant correlation between the volume of search keywords for rubella and measles with monthly reported rubella and measles cases from the Centers for Disease Control and Prevention as well as from the European Center of Disease and Prevention. [30]. Our findings indicate increasing interest among the general public regarding measles and, to a lesser extent, rubella, supporting several traditional epidemiological studies assessing a substantial global resurgence of measles cases [31-33]. The overall outbreak risk for rubella is thought to remain low [31]. The decline in vaccination rates, driven by vaccine hesitancy and the lack of confidence in vaccines, has led to recent outbreaks of vaccine-preventable diseases and threatens the public health gains made against these infectious diseases over past decades [34,35]. These outbreaks are possibly further propagated by travel and migration [33]. Infodemiology data suggest that measles may be of particular concern for some African countries (Niger, Chad, Congo-Kinshasa, and Madagascar), supporting Centers for Disease Control and Prevention notices reporting that some parts of Africa are reporting outbreaks of measles [36]. Ongoing political instability, conflict, lack of education, and poverty may also serve as major barriers to the success of vaccination programs.

Varicella still represents the most widespread vaccine-preventable childhood infectious disease in industrialized countries. Due to its relevant burden on health care resources, several countries have introduced varicella vaccination into the recommended routine childhood national immunization schedule. To date, there has been evidence showing a substantial decline in the varicella incidence from some countries that have introduced varicella vaccination [37-41], but most countries have no data about the impact of vaccination. Unexpectedly, some populations (ie, Republic of Korea) that have implemented universal varicella vaccination are facing increases in the incidence of the disease, possibly explained by primary or secondary vaccine failure [42]. Despite the existence of the varicella vaccine, many resource-rich countries have not implemented routine childhood varicella vaccination into their national schedules [42] partly because of concerns about whether herpes zoster will increase due to a lack of exogenous boosting. Our data show a worldwide increased internet interest for varicella. This finding is consistent with epidemiological studies showing that varicella incidence rates, while unreliable in the absence of mandatory reporting, tend to increase, possibly due to greater urbanization and population density [43].

Our data also support the resurgence of HFMD, more specifically in East Asia. Since 1998, HFMD has emerged, becoming a major public health concern across the Asia-Pacific region. Although the disease is responsible for over 2 million hospitalizations in Asia annually, sporadic outbreaks have occurred in Europe and in the United States in recent years [44].

The reason(s) why genital herpes decreased during this period is unclear but is in line with epidemiological studies showing that herpes simplex virus type 1 and 2 seroprevalences have had a strong declining trend for at least 2 decades, in both sexes and in all different ethnicities, possibly reflecting improvements in hygiene and living conditions [45].

We found nearly no change (slopes) in interest for molluscum contagiosum. The interest for *Pityriasis rosea* decreased over the study period. Although *Pityriasis rosea* is not uncommon, information regarding its global epidemiology is limited. Our findings may support some data assessing that the incidence of *Pityriasis rosea* may be decreasing [46].

Seasonality is a long-recognized attribute of many viral infections, but the mechanisms underlying seasonality, particularly for person-to-person communicable diseases, remain poorly understood. Seasonality may reflect oscillatory changes in infectiousness, contact patterns, pathogen survival, or host susceptibility. Google Trends has been shown to be suitable for studying seasonal patterns of various skin problems including infectious diseases or conditions such as hair loss [47-52], but few studies have used eHealth tools to assess the seasonal variations of a cutaneous viral infection.

Clear seasonality could be observed for some infections. HFMD, varicella, measles, molluscum contagiosum, and warts displayed the highest seasonality coefficients. Peaks of interest were noted in July for HFMD, which is in line with traditional epidemiologic studies showing an association between high temperatures and HFMD [53]. The possible seasonality of erythema infectiosum has been poorly investigated. Confirming classic epidemiological studies, the analysis of big data indicate that varicella also has a distinct seasonal pattern [54]. However, although classic epidemiologic studies suggest that the highest incidence of varicella [55] occurs in winter and spring, internet searches show a peak of interest somewhat later, between April and May. Confirming previous infodemiologic data, we found that the worldwide molluscum contagiosum and wart series showed clear seasonality, with a consistent 12-month oscillation period [56]. These results about seasonal patterns should be interpreted with caution, since we analyzed the worldwide interest in cutaneous viral infections including the Southern and Northern Hemispheres. This bias is probably minimized, since 90% of the world’s population and most of the internet users live in the Northern Hemisphere [22,23]. Viral skin diseases’ seasonality might arise independently of disease incidence, and behavioral trends in skin exposure and self-interest could influence knowledge-seeking for abnormalities such as warts [56].

The main strengths of this study encompass the basic definition of big data, including the “3 V’s”: volume (data sets with large and ever increasing number of observations), variety (the linkage of many structured and unstructured data into a single data set), and velocity (real-time or frequent data updates that are fully automated) [4,13]. Google Trends supports transparency and credibility because these data are openly available and are not limited by complications related to privacy [57], making the analyses replicable by any other investigators. Further, Google Trends topic queries encompass broad literature search terms, search volume data access has remained continuously available since 2008 [58], and the search is not restricted by language.

There are some limitations associated with this analysis. Google Trends provides only an RSV index, not the absolute search volume, and does not provide a way to calculate the search volume index. Google Trends also only provides data related to the selected search terms. Although we chose search terms as inclusively as possible, people looking for information on Google may have chosen other terms. The motivation of Google users is not known. As a corollary, the data obtained from Google Trends cannot be independently verified. The spike of internet searches may be attributed to various factors. It may be due to changes in case numbers in the community and changes driven by government agencies’ announcements, educational purposes, or media coverage (newspapers and newscasts), influencing the web-based research of the population and leading to concerns about the increased risk of obtaining false-positive results [59,60]. Another limitation is that the participant sample was biased toward a certain segment of the population—those with internet access and using Google Search instead of other search engines. However, this bias is mitigated by the fact that as of March 2015, Google accounted for an estimated 65% of all internet search traffic, whereas the next most popular search engine accounted for only 20% of traffic during a given month [61]. Although it is common among the whole population to make web-based health-related searches, younger people tend to use the internet more often. There is also a lack of detailed information on the algorithms Google Trends uses to analyze this search data. For instance, RSV values show a high dependence on the day they are gathered, which can invalidate the reliability of a data set [57]. The collection of worldwide RSVs might minimize these oscillations [57]. It remains also possible that the large amount of data does not necessarily eliminate sources of systematic error and may even amplify them. Finally, Google Trends changed the geographic assignment for internet searches on Jan 1, 2011, and data collection in 2016, which was reflected by Google Trends displaying a respective note in the displayed graphs, but no further information was given [58].

In conclusion, this is the first study using an open web-based infoveillance tool, suggesting that although the incidence of non–vaccine-preventable skin infections is not changing or may be declining, the incidence of vaccinable diseases such as measles, rubella, and varicella is increasing worldwide. The surprising reemergence of measles and other diseases can be attributed to the rise of the anti-vaccination movement, in which groups of people refuse to be vaccinated or have their children vaccinated either out of fear, misinformation, or personal beliefs. The huge potential of the approach of analyzing Google search data could be used in the immediate future to generate timely alerts for clinical epidemiologists on disease outbreaks much earlier than conventional health epidemiology.

## Appendix

Multimedia Appendix 1 Technical details concerning the statistical model and analysis.

## Abbreviations

HFMD: hand, foot, and mouth disease
RSV: relative search volume
