# Supplementary material for: Worldwide Evolution of Vaccinable and Nonvaccinable Viral Skin Infections: Google Trends Analysis
Source: JMIR Dermatol. 2022 Oct 4;5(4):e35034. doi: 10.2196/35034 (PMC10334945; doi:10.2196/35034)
Supplement: Multimedia Appendix 1 [file derma_v5i4e35034_app1.doc]

**Multimedia Appendix 1**

**I. Theoretical aspects**

**Statistical Model**

Time series decomposition separates a time series into five components: mean, long-range trend, seasonality, cycle, and randomness.

The adopted model is a multiplicative model. So the decomposition model is

Value = (Mean) x (Trend) x (Seasonality) x (Cycle) x (Random)

The basic decomposition method consists of estimating the five components of the model

Xt = U Tt Ct St Rt

Where

Xt denotes the series or, optionally, log of series.

U denotes the mean of the series.

Tt denotes the linear trend.

Ct denotes cycle.

St denotes season.

Rt denotes random error.

(t denotes the time period)

**Trend and slope**

In order to calculate the trend,

*We remove the Mean*

As Yt = Xt / Ut

Mt =∑ Yt

*We calculate the Trend*

Mt = a + bt + et

b is the slope.

**Fitting of the model:**

It is evaluated through the **Pseudo R-Squared**

This value generates a statistic that acts like the R-Squared value in multiple regression. A value near zero indicates a poorly fitting model, while a value near one indicates a well-fitting model. The statistic is calculated as follows:


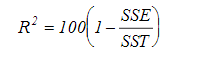


SSE is the sum of square residuals and SST is the total sum of squares after correcting for the mean

**Seasonality**

The seasonality is computed by dividing the Y series by the moving averages.

K_t_= Y_t_ / M_t_

To calculate the seasonal component for each season, we simple average all like seasons.

This is stated as

S_g_= ∑ K_t_

where the summation is over all t in which the season is g.

**There were no missing values.**

*Reference : NCSS Statistical SoftwareNCSS. © NCSS, LLC.*

**II. Details of the analyses under NCSS**

**Seasonal - Trend Report**

Dataset _Mal virales infantiles avec zona et herpès.NCSS

**Forecast Summary Section**

Variable chickenpox

Number of Rows 209

Mean 65,4689

Pseudo R-Squared 0,906004

Mean Square Error 19,56659

Mean |Error| 3,18755

Mean |Percent Error| 5,085708

Forecast Method Winter's with multiplicative seasonal adjustment.

Search Iterations 399

Search Criterion Mean Square Error

Alpha 0,9999936

Beta 2,739309E-10

Gamma 0,06933051

Intercept (A) 61,05183

Slope (B) -0,09137056

Season 1 Factor 1,025033

Season 2 Factor 1,045981

Season 3 Factor 1,162734

Season 4 Factor 1,178287

Season 5 Factor 1,167907

Season 6 Factor 1,134935

Season 7 Factor 0,9499301

Season 8 Factor 0,7934882

Season 9 Factor 0,7661741

Season 10 Factor 0,8382655

Season 11 Factor 0,9272566

Season 12 Factor 1,010008

**Forecast Summary Section**

Variable herpes

Number of Rows 209

Mean 78,64115

Pseudo R-Squared 0,673125

Mean Square Error 16,05924

Mean |Error| 2,987585

Mean |Percent Error| 3,773129

Forecast Method Winter's with multiplicative seasonal adjustment.

Search Iterations 284

Search Criterion Mean Square Error

Alpha 0,5

Beta 0

Gamma 0

Intercept (A) 86,47552

Slope (B) -0,1120981

Season 1 Factor 1,001516

Season 2 Factor 1,027325

Season 3 Factor 0,9994307

Season 4 Factor 1,000794

Season 5 Factor 0,991429

Season 6 Factor 0,9726085

Season 7 Factor 1,029728

Season 8 Factor 1,051099

Season 9 Factor 0,9947239

Season 10 Factor 0,9759802

Season 11 Factor 0,9867743

Season 12 Factor 0,9685901

**Forecast Summary Section**

Variable HFMD

Number of Rows 209

Mean 18,50718

Pseudo R-Squared 0,544182

Mean Square Error 72,64346

Mean |Error| 4,131521

Mean |Percent Error| 23,78003

Forecast Method Winter's with multiplicative seasonal adjustment.

Search Iterations 265

Search Criterion Mean Square Error

Alpha 0,5

Beta 0

Gamma 0

Intercept (A) 15,48409

Slope (B) -0,02411168

Season 1 Factor 0,5521476

Season 2 Factor 0,580572

Season 3 Factor 0,6714981

Season 4 Factor 0,8240315

Season 5 Factor 1,200476

Season 6 Factor 1,216971

Season 7 Factor 1,559607

Season 8 Factor 1,320315

Season 9 Factor 1,146312

Season 10 Factor 1,161163

Season 11 Factor 1,012603

Season 12 Factor 0,7543046

**Forecast Summary Section**

Variable Measles

Number of Rows 209

Mean 24,04785

Pseudo R-Squared 0,620887

Mean Square Error 73,41649

Mean |Error| 4,709287

Mean |Percent Error| 16,50624

Forecast Method Winter's with multiplicative seasonal adjustment.

Search Iterations 263

Search Criterion Mean Square Error

Alpha 0,7176161

Beta 9,650819E-11

Gamma 9,70547E-09

Intercept (A) 12,89881

Slope (B) 0,01480541

Season 1 Factor 0,9228491

Season 2 Factor 1,130336

Season 3 Factor 1,136471

Season 4 Factor 1,284674

Season 5 Factor 1,253132

Season 6 Factor 0,980456

Season 7 Factor 0,8363556

Season 8 Factor 0,9035158

Season 9 Factor 0,9603999

Season 10 Factor 0,8966409

Season 11 Factor 0,9254264

Season 12 Factor 0,7697424

**Forecast Summary Section**

Variable Molluscumcontagiosum

Number of Rows 209

Mean 65,00957

Pseudo R-Squared 0,712029

Mean Square Error 28,52701

Mean |Error| 3,818509

Mean |Percent Error| 5,841094

Forecast Method Winter's with multiplicative seasonal adjustment.

Search Iterations 297

Search Criterion Mean Square Error

Alpha 0,207884

Beta 0,03046034

Gamma 1,015118E-10

Intercept (A) 95,2063

Slope (B) -0,1462657

Season 1 Factor 0,9311768

Season 2 Factor 0,9345309

Season 3 Factor 0,9809416

Season 4 Factor 1,057615

Season 5 Factor 1,124451

Season 6 Factor 1,152938

Season 7 Factor 1,128607

Season 8 Factor 1,075357

Season 9 Factor 0,9903048

Season 10 Factor 0,9118591

Season 11 Factor 0,8952477

Season 12 Factor 0,8169708

**Forecast Summary Section**

Variable PityriasisrosÃ_deGibert

Number of Rows 209

Mean 61,32536

Pseudo R-Squared 0,652280

Mean Square Error 24,66293

Mean |Error| 3,443599

Mean |Percent Error| 5,538779

Forecast Method Winter's with multiplicative seasonal adjustment.

Search Iterations 470

Search Criterion Mean Square Error

Alpha 0,119098

Beta 1,972787E-06

Gamma 2,315217E-10

Intercept (A) 74,44386

Slope (B) -0,1214027

Season 1 Factor 1,025347

Season 2 Factor 1,080184

Season 3 Factor 1,070981

Season 4 Factor 1,06002

Season 5 Factor 1,035503

Season 6 Factor 1,040041

Season 7 Factor 0,9362863

Season 8 Factor 0,8954294

Season 9 Factor 0,9307874

Season 10 Factor 0,9679417

Season 11 Factor 0,984835

Season 12 Factor 0,9726435

**Forecast Summary Section**

Variable Roseola

Number of Rows 209

Mean 61,12918

Pseudo R-Squared 0,811990

Mean Square Error 34,94423

Mean |Error| 4,232054

Mean |Percent Error| 7,232688

Forecast Method Winter's with multiplicative seasonal adjustment.

Search Iterations 292

Search Criterion Mean Square Error

Alpha 0,5

Beta 0

Gamma 0

Intercept (A) 40,4513

Slope (B) 0,1040609

Season 1 Factor 0,8495619

Season 2 Factor 0,9133555

Season 3 Factor 0,9238837

Season 4 Factor 1,044747

Season 5 Factor 1,157517

Season 6 Factor 1,204335

Season 7 Factor 1,244489

Season 8 Factor 1,104248

Season 9 Factor 0,9519285

Season 10 Factor 0,9078204

Season 11 Factor 0,8542629

Season 12 Factor 0,843851

**Forecast Summary Section**

Variable rubella

Number of Rows 209

Mean 40,36842

Pseudo R-Squared 0,635971

Mean Square Error 50,33807

Mean |Error| 4,623206

Mean |Percent Error| 10,56357

Forecast Method Winter's with multiplicative seasonal adjustment.

Search Iterations 241

Search Criterion Mean Square Error

Alpha 0,9783148

Beta 3,558772E-10

Gamma 0,01406215

Intercept (A) 32,28136

Slope (B) -0,01649649

Season 1 Factor 0,906937

Season 2 Factor 0,9988828

Season 3 Factor 1,0769

Season 4 Factor 1,127228

Season 5 Factor 1,147159

Season 6 Factor 1,014218

Season 7 Factor 0,9235795

Season 8 Factor 0,9607139

Season 9 Factor 0,9884388

Season 10 Factor 1,019337

Season 11 Factor 0,9788939

Season 12 Factor 0,8577129

**Forecast Summary Section**

Variable Warts

Number of Rows 209

Mean 68,13397

Pseudo R-Squared 0,965090

Mean Square Error 6,901869

Mean |Error| 1,932618

Mean |Percent Error| 2,977643

Forecast Method Winter's with multiplicative seasonal adjustment.

Search Iterations 442

Search Criterion Mean Square Error

Alpha 0,3111911

Beta 2,217624E-07

Gamma 4,268404E-10

Intercept (A) 54,10942

Slope (B) 0,1366324

Season 1 Factor 0,9224555

Season 2 Factor 0,9255669

Season 3 Factor 0,9508982

Season 4 Factor 1,014396

Season 5 Factor 1,070174

Season 6 Factor 1,110736

Season 7 Factor 1,163047

Season 8 Factor 1,136592

Season 9 Factor 1,024522

Season 10 Factor 0,9398464

Season 11 Factor 0,9025501

Season 12 Factor 0,8392164

**Forecast Summary Section**

Variable zoster

Number of Rows 209

Mean 74,08134

Pseudo R-Squared 0,896369

Mean Square Error 17,53873

Mean |Error| 2,829931

Mean |Percent Error| 3,936787

Forecast Method Winter's with multiplicative seasonal adjustment.

Search Iterations 397

Search Criterion Mean Square Error

Alpha 0,6640668

Beta 0,0002075752

Gamma 8,582307E-10

Intercept (A) 72,51987

Slope (B) 0,09939196

Season 1 Factor 0,9698624

Season 2 Factor 0,9604655

Season 3 Factor 0,9831175

Season 4 Factor 1,004937

Season 5 Factor 1,003237

Season 6 Factor 1,041264

Season 7 Factor 1,045916

Season 8 Factor 1,051712

Season 9 Factor 1,019218

Season 10 Factor 1,010241

Season 11 Factor 0,9825374

Season 12 Factor 0,9274908

**III.Reference**

*NCSS 10 Statistical Software (2015). NCSS, LLC. Kaysville, Utah, USA, ncss.com/software/ncss.*
